# Extrusion-Based 3D Printing of Calcium Magnesium Phosphate Cement Pastes for Degradable Bone Implants

**DOI:** 10.3390/ma14185197

**Published:** 2021-09-10

**Authors:** Lisa-Marie Götz, Katharina Holeczek, Jürgen Groll, Tomasz Jüngst, Uwe Gbureck

**Affiliations:** Department for Functional Materials in Medicine and Dentistry, University Hospital Würzburg, Pleicherwall 2, 97070 Würzburg, Germany; lmgoetz@aol.com (L.-M.G.); katharina.holeczek@stud-mail.uni-wuerzburg.de (K.H.); juergen.groll@fmz.uni-wuerzburg.de (J.G.)

**Keywords:** magnesium phosphate cement, extrusion-based 3D printing, degradable implant

## Abstract

This study aimed to develop printable calcium magnesium phosphate pastes that harden by immersion in ammonium phosphate solution post-printing. Besides the main mineral compound, biocompatible ceramic, magnesium oxide and hydroxypropylmethylcellulose (HPMC) were the crucial components. Two pastes with different powder to liquid ratios of 1.35 g/mL and 1.93 g/mL were characterized regarding their rheological properties. Here, ageing over the course of 24 h showed an increase in viscosity and extrusion force, which was attributed to structural changes in HPMC as well as the formation of magnesium hydroxide by hydration of MgO. The pastes enabled printing of porous scaffolds with good dimensional stability and enabled a setting reaction to struvite when immersed in ammonium phosphate solution. Mechanical performance under compression was approx. 8–20 MPa as a monolithic structure and 1.6–3.0 MPa for printed macroporous scaffolds, depending on parameters such as powder to liquid ratio, ageing time, strand thickness and distance.

## 1. Introduction

Synthetic bone replacement materials are usually based on calcium phosphate chemistry with more than 50 years of clinical experience, whereas hydroxyapatite (Ca_10_(PO_4_)_6_(OH)_2_) and ß-tricalcium phosphate (ß-Ca_3_(PO_4_)_2_) are the most prominent available materials [1]. Clinical experience over decades shows good healing results with a direct bonding to bone and an ingrowth of newly formed bone tissue into larger pores [2], however the slow degradation, especially of hydroxyapatite, often leads to the formation of an osseoceramic regenerate instead of a full bony regeneration at the defect site. The reason behind this is the low solubility of such calcium phosphates under in vivo conditions, whereby the degradation kinetics is further influenced by material parameters such as porosity, crystallinity, or phase composition. Degradation in vivo mostly occurs via cellular activity of osteoclasts, which locally lower the pH leading to an increase in calcium phosphate solubility by at least two orders of magnitude [3,4,5]. Since this process is limited to the material surface, a faster degradation requires interconnected pores >100 µm for cell infiltration, which in turn weakens the mechanical material performance due to the well-known relationship between porosity and strength of ceramics [6].

Magnesium phosphate minerals are discussed as suitable alternatives for bone replacement as they provide higher solubility with fast bone regeneration [7,8,9]. Even for low porous cement monoliths composed of struvite (MgNH_4_PO_4_·6H_2_O) and farringtonite (Mg_3_(PO_4_)_2_), the dissolution of struvite within four months leads to tissue ingrowth into the cement and a nearly complete defect regeneration after 10 months in a large animal model [10,11]. Calcium phosphate references such as calcium deficient hydroxyapatite or brushite (CaHPO_4_·2H_2_O) showed only marginal degradation within the same time period. Similar trends were also observed in vitro and in vivo for other magnesium phosphate minerals like newberyite [12], cattiite [13] or calcium substituted MgPs with a composition Mg_(3−x)_Ca_x_(PO_4_)_2_ [14]. The latter are of interest as the Ca:Mg ratio in such materials is a versatile parameter to adjust the osteoclastic resorption profile [15].

In the past, the processing of magnesium phosphates usually occurred in the form of aqueous cement pastes that had to be manually molded into a defect. In contrast to calcium phosphate ceramics [16,17], additive manufacturing (AM) approaches to creating scaffolds or implants are rarely described for MgP minerals. Previous approaches applied 3D powder printing to create 3D structures from cement powder and an aqueous binder solution [18], however this fabrication regime is limited to the processing of monolithic materials and does not offer the possibility to produce composites. This limitation can be overcome by extrusion-based 3D printing, which enables the printing of polymer–ceramic composites [19] and the simultaneous processing of different materials in one scaffold [20] but has not been used to process cold-setting MgP cements yet.

In the present study, we chose extrusion-based 3D printing of MgP cement pastes as an AM technique for scaffold fabrication to expand the fabrication methods for the generation of porous MgP scaffolds. We produced aqueous cement paste inks by dispersing a calcium magnesium phosphate cement powder with the stoichiometric composition Ca_0.75_Mg_2.25_(PO_4_)_2_ and hydroxypropylmethylcellulose (HPMC) in water. These inks are extrinsically set by immersion in ammonium phosphate solution, leading to the formation of the setting product struvite (MgNH_4_PO_4_·6H_2_O). All cement pastes were characterized regarding their rheology and extrusion through 410–610 µm nozzles and mechanical properties after hardening. Finally, 3D printing experiments were performed to evaluate printing quality, dimensional stability, and mechanical performance of the printed scaffolds.

## 2. Materials and Methods

### 2.1. Cement Paste Preparation

Ca_0.75_Mg_2.25_(PO_4_)_2_ as cement raw material was synthesized by sintering mixtures of MgHPO_4_·3H_2_O (Sigma-Aldrich, Schnelldorf, Germany), CaHPO_4_ (Baker, Griesheim, Germany), CaCO_3_ (Merck, Darmstadt, Germany) and Mg(OH)_2_ (VWR, Ismaning, Germany) at an appropriate stoichiometric ratio at 1100 °C for 5 h. The sintered cake was manually crushed, sieved <125 µm and ground dry for 4 h in a ball mill (Retsch PM400, Idar-Oberstein, Germany). Aqueous cement pastes were obtained by mixing 11 g of raw CaMgP powder with 2.5 g of MgO (Magnesia 2933), 1 g of hydroxypropylmethylcellulose (HPMC) powder and 7–10 mL of water. The setting reaction of such pastes was initiated by immersion in 3.5 M (NH_4_)_2_HPO_4_ (DAHP) solution for 24 h to induce struvite precipitation.

### 2.2. Rheological Cement Paste Properties

The rheological investigation of the cement pastes was carried out with a rheometer (MCR301, Anton Paar, Ashland, OR, USA) in plate-plate geometry (diameter 50 mm). For this purpose, the storage modulus G′ and the loss modulus G″ were recorded by the Rheoplus software (Anton Paar, Ashland, OR, USA) in an oscillation test over a period of 7 h at a frequency of 10 1/s and an amplitude of 0.1% deformation. To determine this frequency and amplitude, an amplitude and frequency sweep was performed both at the beginning and at the end of the measurement. Thus, the deflection amplitude was increased at a fixed frequency and in addition the angular frequency was increased at an amplitude lying in the linear viscoelastic range. As a reference, HPMC dissolved in H_2_O was investigated in rotational tests according to different storage times without further components. For this purpose, the viscosity was recorded in a controlled shear rate (CSR) test with increasing shear rate. All measurements were performed at 21.3 °C. A solvent trap was used to protect the cement paste from drying.

### 2.3. Compressive Strength Testing

In order to test the stability of the cements, six rectangular test specimens, each with dimensions 6 mm × 6 mm × 12 mm, were produced. For this purpose, the cement pastes were filled into silicone molds and then placed in a water bath at 37 °C in 3.5 M DAHP solution for 24 h. The test specimens were then removed from the silicone molds and left in DAHP solution for an additional 24 h. Subsequently, the compressive strength of the specimens was determined using the testing machine (Z010, Zwick/Roell, Ulm, Germany) and a 10 kN load cell. For this purpose, the specimens were loaded axially at a crosshead speed of 1 mm/min and the maximum force required for fracture was measured. The mean value and standard deviation were then calculated from each of the six measurements by testXpert2 software (Zwick/Roell, Ulm, Germany). Compression strength of printed samples was tested with the same device as was used for the rectangular specimens. A plunger with a diameter of 7.5 mm was used to test printed samples with a base area of 12 mm × 12 mm. Compression was applied perpendicular to the build platform orientation during printing.

### 2.4. Porosity and Pore Size Distribution

Porosity and pore size distribution was measured on hardened cement fragments by mercury intrusion porosimetry (Porosimeters Pascal 140 and 440, Thermo Fisher Scientific, Monza, Italy). Hardened cement samples were placed in the dilatometer, and after filling with mercury a gradual pressure ranging from 0.01 to 400 kPa was applied, followed by a second measurement at a higher-pressure range of 0.1 to 400 MPa. Both measurements were combined with the associated software “Solid” (Thermo Fisher Scientific, Monza, Italy) to obtain a complete pore size distribution ranging from 10 nm to 100 µm.

### 2.5. In Vitro Degradation in Phosphate-Buffered Saline (PBS) and Simulated Body Fluid (SBF)

PBS with pH = 7.4 was obtained by dissolving 137 mM NaCl, 2.7 mM KCl, 7.0 mM Na_2_HPO_4_·2H_2_O, 1.5 mM KH_2_PO_4_ (all from Merck, Darmstadt, Germany) in deionized water. Hardened cement samples (*n* = 5) with a cylindrical shape (d = 5 mm, h = 3 mm) were immersed in 10 mL PBS buffer. Over the course of 14 days, the medium was exchanged daily and the samples were weighed to determine mass loss. As reference, α-tricalcium phosphate cement similar to the study of Kanter et al. [11] was used. SBF was used to analyze the influence of storage conditions on printed samples after the setting reaction. SBF was prepared according to standard procedures by dissolving 7.995 g NaCl, 0.2237 g KCl, 0.30495 g MgCl_2_ ·6 H_2_O, 0.07102 g Na_2_SO_4_ (all from Merck, Darmstadt, Germany), 0.36755 g CaCl_2_ ·2 H_2_O (Baker, Griesheim, Germany), 0.35285 g NaHCO_3_ (VWR, Ismaning, Germany), and 0.17418 g K_2_HPO_4_ (VWR, Ismaning, Germany) in l liter of milliQ water and the pH was adjusted to 7.4 by the addition of HCl or Trizma base (Sigma, Schnelldorf, Germany). Samples immersed in SBF were stored in a water bath at 37 °C for 20 d. SBF was changed after four and ten days.

### 2.6. 3D Printing Experiments

For three-dimensional printing of the cement pastes, a 3D printer (3D Discovery, regenHU, Villaz-St-Pierre, Switzerland) was used. A print head based on a “time-pressure” principle (DD-135N, 900 002 772, regen HU, Villaz-St-Pierre, Switzerland) in combination with 5 mL and 10 mL cartridges (Nordson EFD, Erkrath, Germany) was chosen to conduct the experiments. For dispensing, compressed air up to 5 bar was utilized to adjust the desired material flow. Conical dispensing tips (cone TT, Nordson EFD, Erkrath, Germany) with different diameters (18, 20, 22, and 27 gauge) were used for extrusion-based printing. Printing was performed at room temperature; no heating or cooling was involved.

### 2.7. Phase Composition

Hardened cement fragments were ground and further analyzed via X-ray diffractometry (XRD, D5005, Siemens, Karlsruhe, Germany) using Cu-K_α_ radiation, a 40 kV voltage, a 40 mA current, a 2 theta range from 10 to 40°, a step size of 0.02° and a scan rate of 1.5 s/step. JCPDS references of stanfieldite (PDF-Nr. 011-0231), struvite (PDF-Nr. 015-0762) and farringtonite (PDF-Nr. 033-0876) were taken into account for XRD pattern evaluation.

For the determination of the quantitative struvite content, a calibration line according to the internal standard method [21] was used. For this purpose, calcium carbonate CaCO_3_ as alibi phase and phase-pure struvite were mixed and calcium fluoride CaF_2_ was added as an internal standard phase at a concentration of 20%. The ratio of the integral peak intensities of struvite at 16.5° and CaF_2_ at 28.3° was then linearly related to the struvite content in wt% according to Equation (1) [21].

x = I1/I2 × k(1)

x = struvite content [wt%]
k = 0.00472 (proportionality constant)
I1 = integrale peak intensity of struvite
I2 = integrale peak intensity of CaF_2_

### 2.8. Morphological Analysis

A stereomicroscope (SteREO Discovery.V20, Zeiss, Jena, Germany) was used to examine the hardened and dried scaffolds through a 1.5 × PWD 30 mm objective (Plan Apo S, Zeiss, Jena, Germany). Photographs were taken for this purpose with a camera integrated in the microscope (Axio Cam ICc5, Zeiss, Jena, Germany) vertically from above at 5×, 10×, and 20× magnification and also laterally at angles of approximately 90° and 45° at 5× magnification. ImageJ image processing software was then used to measure the images with respect to strand thickness and strand spacing. The mean value and standard deviation were calculated from 10 measurements of the strand thickness for each of the six scaffolds.

Scaffold microstructure was analyzed by scanning electron microscopy (SEM) after sputtering with a 4 nm platinum layer (Leica EM ACE600, Leica Microsystems, Wetzlar, Germany) with a scanning electron microscope Crossbeam 340 (SEM, Zeiss, Oberkochen, Germany).

## 3. Results

**Figure 1:**
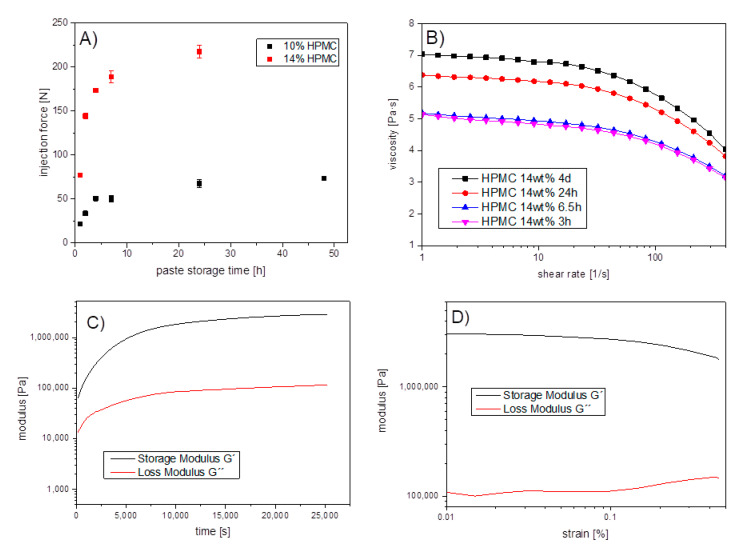
Rheological properties of the fabricated cement pastes: (**A**) medium load for cement paste extrusion from a syringe with 18 G cannula depending on storage time of the pastes; (**B**) time-dependent viscosity of a HPMC solution in water without further cement additives; (**C**) storage- G′ and loss modulus G″ of a 10 wt% HPMC/magnesium phosphate paste in an oscillation regime over 7 h at a frequency of 10 1/s, an amplitude of 0.1% and a temperature of 21.3 °C; (**D**) amplitude sweep of the paste at an amplitude of 0.1% and a temperature of 21.3 °C after 7 h oscillation.

Investigations into cement paste properties for extrusion-based 3D printing started with measuring injectability through an 18 G cannula (Figure 1A), demonstrating the influence of both storage time of the pastes after fabrication and the powder to liquid ratio on extrusion. While the latter is not unexpected, ageing of the pastes over 24 h increased the injection force by 181–247%. Since the change in cement paste viscosity might be related to a time-dependent swelling of the HPMC component, viscosity measurements of HPMC dissolved in pure water were performed (Figure 1B) using a rheometer. The results showed a storage time dependent increase in gel viscosity and shear-thinning properties of the HPMC solution. The storage modulus G′ and loss modulus G″ of the cement pastes were determined over a period of 7 h at a frequency of 10 1/s and an amplitude of 0.1%. As shown in Figure 1C, both G′ and G″ increased steeply until an inflection point of about 1 h 20 min (5000 s), and then steadily approached a plateau value of about 0.1 MPa for G″ and 3 MPa for G′ after 7 h. This indicates that the elastic fraction increased more than the viscous fraction and that the investigated cement paste became increasingly solid with time. Subsequently, an amplitude sweep was performed to check time-dependent material properties (Figure 1D). At 21.3 °C and a frequency of 10 1/s it could be shown that at 0.1% deformation G′ reached higher values than G″, while lg G′ and lg G″ were parallel both to each other and to the deformation axis, and thus the measured values were in the LVE range.

**Figure 2:**
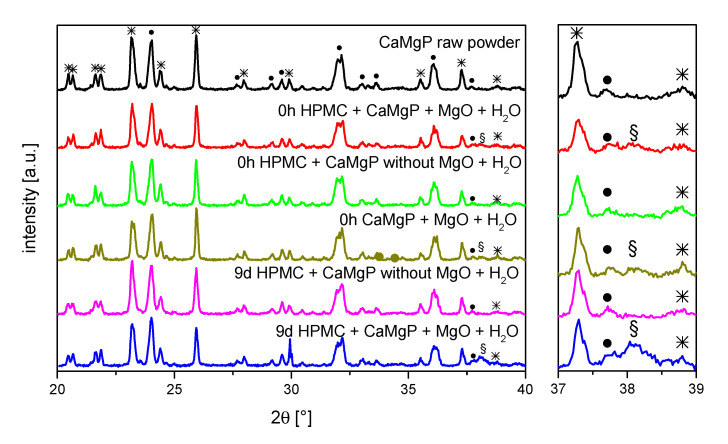
X-ray diffraction patterns of different cement pastes stored for up to 9 d at 37 °C; * farringtonite, • stanfieldite, § most likely brucite.

To identify whether the change in rheology is further related to compositional changes of the cement paste composition, X-ray diffraction patterns were recorded before and after ageing for up to nine days (Figure 2). The only differences observed were an additional diffraction peak at around 38.1° for those pastes that contained MgO, whereas the intensity of the peak increased with storage time. Most likely, this peak can be attributed to brucite (Mg(OH)_2_) formed by hydrolysis of the MgO component. A further reaction of the farringtonite or stanfieldite phases could not be observed.

**Figure 3:**
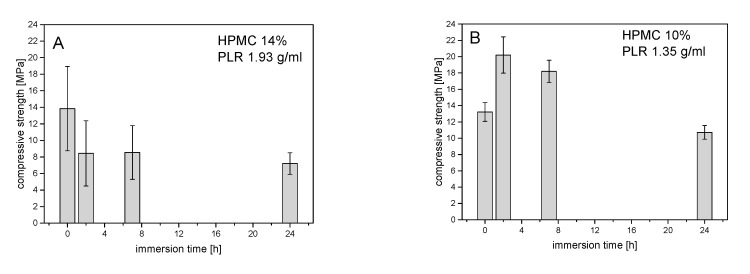
Compressive strength of hardened magnesium phosphate cement pastes with either (**A**) 14 wt.% HPMC (PLR 1.93 g/mL) or (**B**) 10 wt.% HPMC (PLR 1.35 g/mL) depending on the storage time of the pastes at 37 °C prior to hardening.

**Figure 4:**
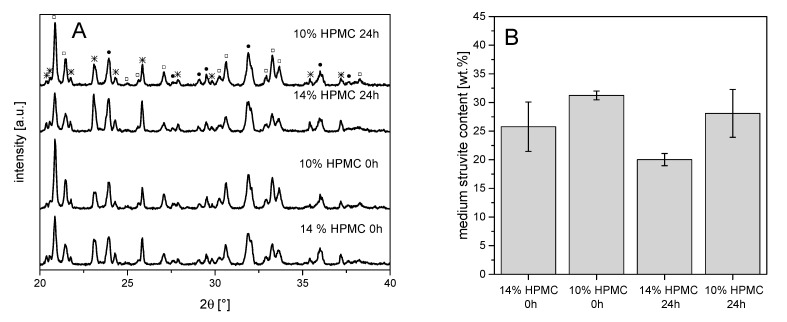
(**A**) X-ray diffraction patterns and (**B**) struvite content of Mg_2.25_Ca_0.75_(PO_4_)_2_ magnesium phosphate cement pastes with different powder to liquid ratios (14% ≙ PLR 1.93; 10% ≙ PLR 1.35) after 0 and 24 h storage of the paste at 37 °C prior to hardening; □: struvite, ●: stanfieldite, *: farringtonite.

In general, compressive strength–storage time diagrams showed compressive strength in the range between 6 and 20 MPa, which became significantly smaller with storage time, as shown in Figure 3. At a powder-to-liquid ratio (PLR) of 1.35 (≙ 10% HPMC), the low compressive strength at 0 h was due to the inaccurate specimen dimensions as the samples were not fully hardened at the bottom side in the molds (Figure 3). Taking this error into account, no clear effect of the H_2_O content on the compressive strength could be detected at different PLRs. The evaluation of the XRDs of the hardened cements in Figure 4 showed an influence of PLR on the struvite content. With a PLR of 1.35 (≙ 10% HPMC), the struvite content was around 30 wt.%, while cements with a PLR of 1.93 (≙ 14% HPMC) contained only between 20 and 25 wt.% struvite.

**Figure 5:**
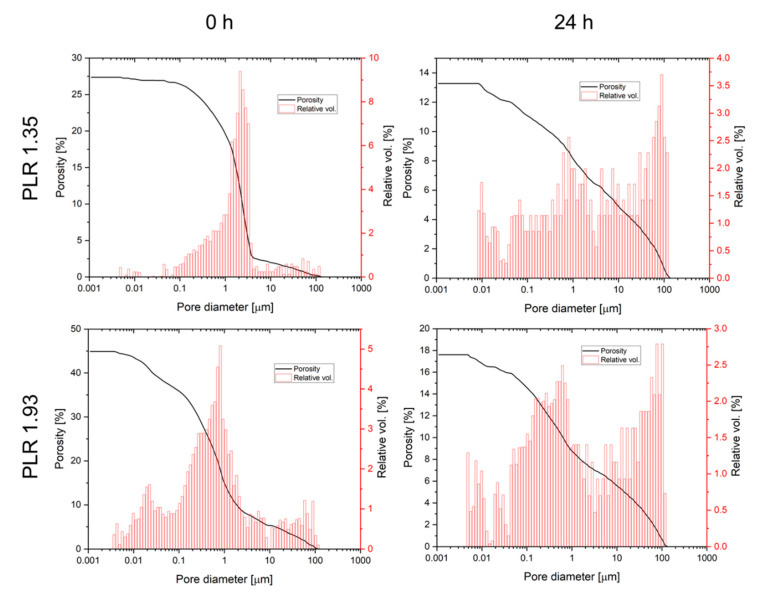
Porosity and pore size distribution of CaMgPO_4_ cement pastes immediately after preparation and after 24 h ageing at 37 °C. Samples were hardened in 3.5 M (NH_4_)_2_HPO_4_ solution for 24 h prior to measurement.

**Figure 6:**
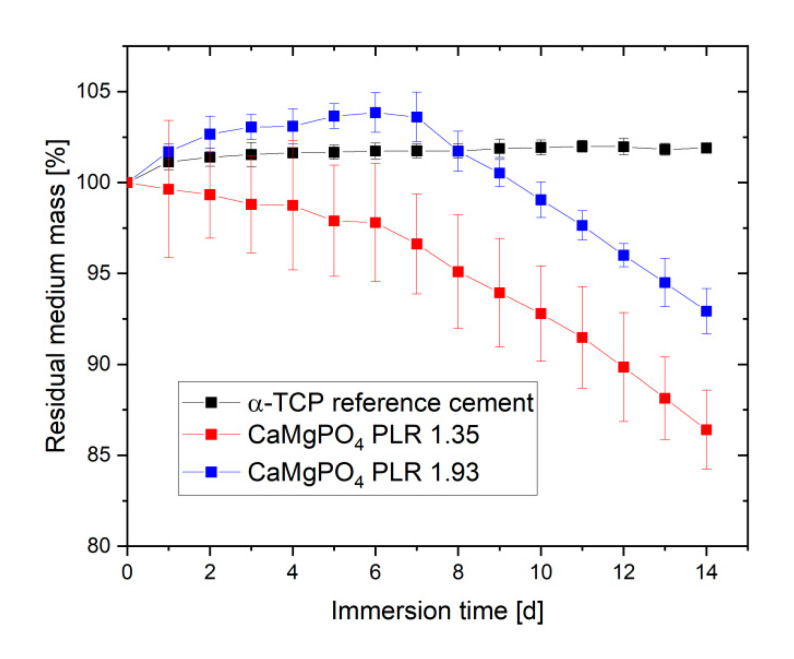
Dissolution profiles of 24 h stored cement pastes, which were hardened in 3.5 M (NH_4_)_2_HPO_4_ solution for 24 h. Samples were immersed in 10 mL PBS buffer at 37 °C over a course of 14 d with a daily change in the immersion liquid (*n* = 5).

Ageing of the pastes also had an effect on both total porosity and pore size distribution after hardening (Figure 5). Total porosity was found to decrease for both pastes from 25–45% directly after preparation to values of 13.1–17.6% after 24 h cement paste storage. In addition, for both pastes the pore size distribution was altered by ageing leading to a loss of pore volume in the 0.5–5 µm range, while the volume of larger pores >10 µm increased. Degradation of cement pastes was studied by immersion in PBS buffer with a daily change of the immersion liquid (Figure 6). While the calcium phosphate reference cement slightly increased weight over the course of the experiment, the magnesium calcium phosphate cements showed a linear degradation profile with a total mass loss ranging from 7% (PLR 1.93) to 14% (PLR 1.35) after day seven. Differences between both PLRs mostly occurred initially, whereas the higher PLR cement firstly showed an increase in mass, most likely related to the water absorption of the HPMC fraction. For the lower PLR, this effect was not observed as this cement contained approx. 30% less HPMC.

**Figure 7:**
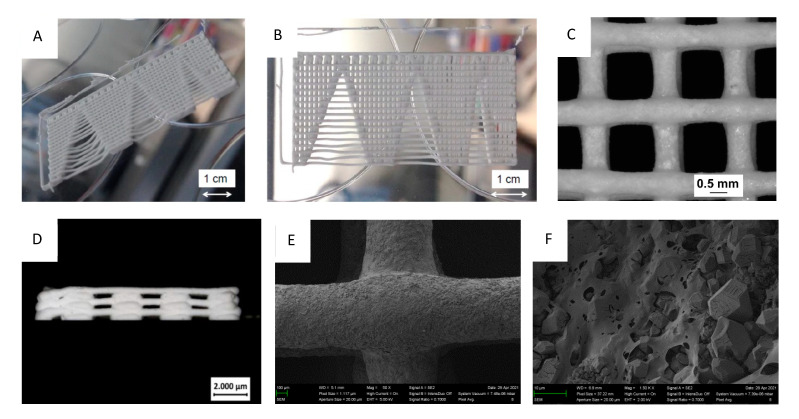
(**A**,**B**) printed structure to determine the maximum distance at which strands become unstable; (**C**,**D**) top and side view stereomicroscopic images of a six-layered 12 × 12 mm scaffold printed with a 0.41 mm cannula and a strand distance of 1.5 mm, (**E**,**F**) SEM images of two crossing strands at different magnifications.

For 3D printing, air pressure at the cartridge and printing speed had to be carefully adjusted to prevent sagging of the individual strands and strand breaking during fabrication. In addition, the right dispensing pressure ensures dimensional fidelity and helps control the fusing of the strands (Figure 7). To investigate the spanning properties of the inks, a test structure (shown in Figure 7A,B) was designed and printed with the ink that is evaluated. The structure contained progressively increasing spanning distances and thus enables us to determine the maximum spanning distance for a test ink by detecting the biggest spanning distance as the shortest distance before the strand touches the collector. For the 0.61 mm cannula, by changing the air pressure, it was shown that up to 5.25 mm greater distances could be bridged at 1 bar pressure.

Scaffolds of 12 mm × 12 mm size with strand spacing of 1.5 mm each were printed both through 0.61 mm and 0.41 mm cannulas and six to eight layers directly in Petri dishes. Furthermore, 2 mm strand spacing at 12 mm × 12 mm size was also printed through 0.61 mm cannulas and 1 mm strand spacing at 10 mm × 10 mm size was printed through 0.41 mm cannulas. Figure 7C shows the microscopic images of mentioned scaffolds at 5× magnification, with strands flattened into each other at the corner of the printing start as well as at the opposite corner of the scaffold. A measurement of the individual paste strands showed negligible deviations between the diameter of the cannula and the thickness of the strands of the scaffolds. At 0.61 mm cannula diameter, the mean value of all measurements was (0.62 ± 0.02) mm, while at 0.41 mm cannula diameter it was (0.38 ± 0.01) mm. A side view of the scaffold also demonstrated high printing quality in the z-direction with only a flattening of the strands at the left corner where the printing process started (Figure 7D). SEM images (Figure 7E,F) reveal a gap-free interface between crossing strands. In addition, the microstructure is characterized by a relatively low porous cement texture with irregular shaped cement particles embedded in the polymeric matrix.

**Figure 8:**
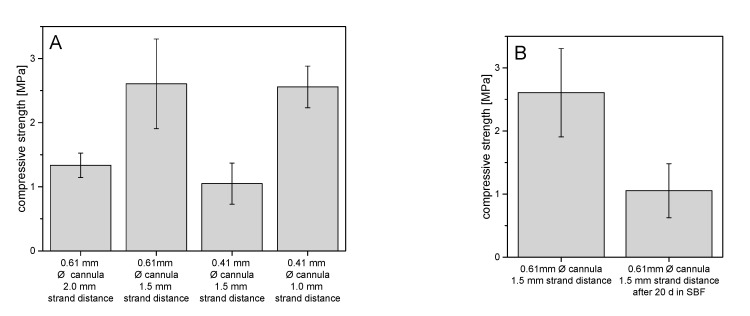
Compressive strength of printed scaffolds (**A**) with variation of strand distance and cannula diameter and (**B**) before and after immersion in SBF solution for 20 days.

The stabilities of the printed struvite scaffolds were compared on the basis of their mechanical performance under compression (Figure 8). Overall, the strengths of all scaffolds were between about 1 MPa and 2.5 MPa. It could be seen that with a 0.5 mm decrease in strand spacing, but no change in cannula diameter, the strength increased by about 1.3 MPa to 2.5 MPa. When the cannula diameter decreased by 0.2 mm but the strand spacing remained the same at 1.5 mm, the strength decreased by about 1.6 MPa, as expected. In Figure 8B, scaffolds of the same architecture of 0.61 mm strand thickness and 1.5 mm strand spacing, but with and without 20-day aging, are compared on the basis of their strengths under compression. After aging in simulated body fluid (SBF), the scaffolds had strengths of about 1.0 MPa, which is about 1.6 MPa lower than the non-aged scaffolds.

## 4. Discussion

Within this study we were able to develop printable calcium magnesium phosphate pastes, which could be hardened by immersion in ammonium phosphate solution. The crucial components were—besides the main mineral compound as biocompatible ceramic—magnesium oxide and hydroxypropylmethylcellulose. While MgO was necessary to achieve rapid hardening post-printing, HPMC was needed to increase paste viscosity to ensure that the printed samples maintained their shape during the printing process. This enabled printing even through 0.41 mm cannulas with a sufficient dimensional stability of the printed strands.

However, during our work we found a subsequent viscosity increase during storage of the freshly prepared cement pastes. A structural change of HPMC as a component of cement paste was considered the cause of viscosity change with storage time. Thus, rheological tests of HPMC dissolved in H_2_O were conducted at specific time points between 3 h and 4 d. In a CSR test at different storage times, it was confirmed that the viscosity increased with the storage time of HPMC solutions. Furthermore, it was demonstrated that the viscosity decreased with increasing shear rate (see Figure 1B). As expected for a long-chain and unbranched molecule, HPMC in H_2_O was a shear-thinning material, which is consistent with the results of the rheological studies by Fatimi et al. [22]. Increasing entanglement of polymer chains with storage time resulted in an increase in viscosity. At higher shear rates these polymer networks disentangle and the polymer chains align in the shear stress direction resulting in a drop in viscosity.

Another influencing factor for the observed viscosity increase with storage time could be a reaction of the mineral components of the paste. XRD examinations of the pastes directly after production and after a time interval of 9 d identified a Mg(OH)_2_ peak at 38.1°, which was much more pronounced after 9 d storage than directly after mixing of the pastes. Since such a peak was not found in pastes without MgO addition, it is reasonable to assume that Mg(OH)_2_ has been formed by reaction of H_2_O with MgO (Equation (2)) [23,24]. Here, it is quite conceivable that the formation of the hydrated phase led to stronger attractive interparticular interactions in the paste, causing an increase in viscosity.

MgO + H_2_O ⇌ Mg^2+^ + 2 OH^−^ ⇌ Mg(OH)_2_ (2)

Hardening of the pastes was initiated by immersion in ammonium phosphate solution, whereas initially magnesium oxide (or the hydrated form) reacts, leading to rapid hardening (Equation (3)), followed by the reaction of farringtonite according to Equation (4). Ammonium hydroxide and phosphoric acid formed as side products of both reactions will instantly react in an acid–base reaction to form primary ammonium phosphate (5), which can again participate in the setting reaction to struvite:

MgO + (NH_4_)_2_HPO_4_→2MgNH_4_PO_4_ ·6H_2_O + NH_4_OH (3)

Mg_3_(PO_4_)_2_ + (NH_4_)_2_HPO_4_→2MgNH_4_PO_4_ ·6H_2_O + H_3_PO_4_ (4)

H_3_PO_4_ + NH_4_OH→NH_4_H_2_PO_4_ + H_2_O (5)

The storage time of the pastes also had an influence on the properties of the cements set to struvite. For all cement pastes, the struvite content observed in the XRD tended to be lower after 24 h than after 0 h storage time. This decrease in struvite content with storage time was due to the described increase in viscosity, which impedes diffusion of DAHP into the paste and thus leads to a more incomplete setting reaction with lower struvite content. Furthermore, the lower struvite content also had a negative effect on the compressive strength of the struvite specimens. As the storage time of the pastes increased, the compressive strength decreased, which was similar to the reverse of the viscosity increase during the injection tests. Although the pastes were stored in a large stoichiometric excess of DAHP, no quantitative conversion to struvite occurred. The reason for this is probably a decreasing microporosity of the cement structure with increasing conversion since the precipitating struvite with its low density strongly confines the pore volume. This is shown, for example, in investigations by Kanter et al. [11], who found a porosity of only 5–7% for struvite cements depending on the PLR. This low microporosity in combination with the swollen HPMC phase in turn prohibits diffusion of ammonium and phosphate ions into the structure and hence inhibits further struvite formation.

Using the developed pastes enabled printing of scaffolds with a sufficient dimensional accuracy as shown in Figure 7. This was not only the case in the xy-direction but also visible from the side view for printing in the z direction. While the use of smaller cannulas may further improve printing accuracy, clogging of such small cannulas may interfere with the overall reproducibility of the whole printing process according to our experience. In addition, smaller strands with a similar distance also had negative effects on the mechanical performance of the scaffolds. The mechanical performance of the printed scaffolds was in the range of 1–2.5 MPa under compression. Here, it became clear that a smaller strand spacing and a larger cannula diameter provided more fracture resistant scaffolds, as expected and as already shown by Lee et al. [25]. This fact occurred because of both smaller inter-strand spacing and thicker strand minimizing voids within the overall scaffold volume. Thus, for the same test area, there was more material to resist the force of the testing machine, resulting in higher strengths. The strength is thus comparable to printed calcium phosphate cement scaffolds [26] and should be sufficient to ensure clinical handling, although appropriate mechanical stabilization by metallic osteosynthesis systems would be required for larger defects. The advantage of the developed magnesium phosphate scaffolds would be better solubility compared to calcium phosphates and thus faster degradation behavior as shown by our dissolution experiment in PBS buffer (Figure 6). Interestingly, linear fitting of both curves after day seven resulted in an estimated total dissolution time of 73–76 days for both cements, independent of the powder to liquid ratio. However, such a linear degradation profile is somewhere speculative since the scaffolds have a biphasic phase composition of struvite and remaining farringtonite raw material according to XRD investigations (Figure 4). The in vivo resorption of such materials is known to be a two-step process, in which the struvite cement component is initially dissolved until month four, followed by the dissolution of the farringtonite cement component until month seven [11]. In addition, material degradation in vivo not only occurs by passive dissolution but also by the cellular activity of osteoclasts [5]. The latter produce a local acidic environment by secretion of protons, which increases solubility of phosphate salts by at least two orders of magnitude. This was investigated for a variety of phosphate minerals by Grossardt et al. [27] and Blum et al. [15] by culturing multinuclear osteoclastic cells derived from RAW 264.7 macrophages on the materials surface. Both studies revealed that magnesium phosphate mineral degradation predominantly occurs by osteoclast activity with a total resorption of approximately 4.7% during the course of 13 days. These data from the literature suggest that the printed scaffolds from our study are also thought to show fast bone regeneration similar to aqueous magnesium phosphate cement pastes with comparable composition [10].

## 5. Conclusions

The current approach of printing magnesium phosphate cement pastes used an extrinsic setting mechanism by immersion of the scaffolds in ammonium phosphate solution. Although this was successful at the first attempt, some disadvantages remain, for example ageing effects of the cement on paste viscosity and an incomplete conversion to struvite as the rapidly degradable mineral phase. Here, further modifications might be possible to overcome these problems like combining both the cement powder and ammonium phosphate in the paste using a non-aqueous but water miscible carrier liquid such as glycerol or low molecular weight polyethylenglycol. This would enable an intrinsic hardening by diffusion of water into the paste and—by adjusting stoichiometry—would also help to increase the degree of conversion into struvite. Furthermore, the hardening of such pastes might be directly initiated during printing, e.g., by actively mixing with water or by using a coaxial printing approach as recently described for calcium phosphate cements [28]. This would be beneficial for the fabrication of larger implants, which otherwise might collapse during printing due to their own weight.

## Data Availability

Data sharing is not applicable to this article.
